# Size-Dependent Persistent Luminescence of YAGG:Cr^3+^ Nanophosphors

**DOI:** 10.3390/ma15134407

**Published:** 2022-06-22

**Authors:** Vitalii Boiko, Zhengfa Dai, Mykhailo Chaika, Karina Grzeszkiewicz, Jiang Li, Wieslaw Strek, Dariusz Hreniak

**Affiliations:** 1 Division of Optical Spectroscopy, Institute of Low Temperature and Structure Research, Polish Academy of Sciences, Okolna 2, PL 50-422 Wroclaw, Poland; m.chaika@intibs.pl (M.C.); k.grzeszkiewicz@intibs.pl (K.G.); w.strek@intibs.pl (W.S.); d.hreniak@intibs.pl (D.H.); 2 Key Laboratory of Transparent Opto-Functional Inorganic Materials, Shanghai Institute of Ceramics, Chinese Academy of Sciences, Shanghai 201899, China; daizhengfa@mail.sic.ac.cn (Z.D.); lijiang@mail.sic.ac.cn (J.L.); 3 Center of Materials Science and Optoelectronics Engineering, University of Chinese Academy of Sciences, Beijing 100049, China

**Keywords:** YAGG, transition metal (Cr), lattice parameter, persistent luminescence, thermoluminescence, traps redistribution

## Abstract

In the current work, YAGG:Cr^3+^ nanophosphors were synthesized by the Pechini method and then annealed at different temperatures in the range 800–1300 °C. The structure and morphology of the samples were characterized by X-ray Powder Diffraction (XRPD). The lattice parameters and average crystalline sizes as site occupation by Al^3+^ and Ga^3+^ ions were calculated from the Rietveld refinement data. To investigate the effect of crystalline size of the materials on their optical properties: excitation and emission spectra were recorded and analyzed. Finally, the effect of crystalline size on the probability of carrier recombination leading to PersL was determined experimentally with thermoluminescence analyses. The T_max_-T_stop_ method was applied to determine the trap type and particle size (calcination temperature) effect on their redistribution. A correlation between structural changes and trap redistribution was found. In particular, the extinction of high-temperature TL maximum with increasing annealing temperatures is observed, while low-temperature TL maximum increases and reaches a maximum when the lattice parameter reaches saturation.

## 1. Introduction

The recently observed increase in interest in nanomaterials that could combine luminescent properties with the ability of temporary storage and emission release due to thermal (thermoluminescence, TL) and optical (optically stimulated luminescence, OSL) stimuli has resulted in the numerous publications on so-called persistent nanophosphors [1,2,3,4,5,6,7]. Due to potential biomedical applications, the most studied compounds are chemically stable oxides with spinel and garnet structures that provide better biocompatibility compared to, for example, fluorides and sulfides. In these materials, specific positions in the crystal lattice are defined, which can be substituted in a controlled way with acceptor and donor ions involved in the mechanism of electron and hole trapping, as well as their release leading to radiative deactivation [2,8]. An additional advantage of garnet and spinel structured materials is the ability to simultaneously control the energy gap value of the host material by introducing appropriate concentrations of other cations [9].

Ceramics and micro and nanocrystals based on the garnet matrix are the most perspective oxide crystals widely used as the active medium in solid-state lasers. Due to the anisotropic optical properties, YAG powders doped with rare-earth (Ce^3+^, Pr^3+^, Yb^3+^, and Nd^3+^) and transition metals (Mn^2+^, Cr^3+^, etc.) are suitable for preparing transparent laser ceramics with the working region in the near-infrared range that is suitable for bio-applications [10]. At the same time, several publications have described the modulation of the band gap by replacing Ga^3+^ with Al^3+^ in the Y_3_Al_5-x_Ga_x_O_12_ matrix to obtain Y_3_Al_2_Ga_3_O_12_ (YAGG) stoichiometry, which has been shown to be optimal for use as garnet PersL host material [9,11,12]. As a result, YAGG:Cr co-doped with lanthanides have gained attention due to their interesting persistent luminescence (PersL) in the visible, red, and NIR range, where Cr ions play an important role in obtaining long-lasting emissions.

Unfortunately, the theoretically very predictable results of the above actions for optimizing persistent luminescence (PersL) quality (intensity and duration) are perturbed in nanoscale particles due to structural imperfections related to size effects as well as the greater influence of the environment of these particles on radiative and non-radiative processes occurring in their volume [13]. Additionally, garnet powder is a convenient material for the manufacture of laser and persistent luminescence ceramics from nanosized grains by low-temperature sintering under high pressure [14,15]. Furthermore, the duration and color of PersL are determined by the luminescence center. Therefore, the use of Cr ions both as an independent dopant and as a co-dopant extends the emission time for most of the used matrices and shifts the emission range to the NIR region [9,16,17,18].

In this work, we present the results of a spectroscopic study that allow us to better understand the influence of these effects using a model system of Y_3_Al_2_Ga_3_O_12_ garnet (YAGG) doped only with chromium ions, which, according to previously accepted models, act both as dopants leading to the formation of trapping centers and leading to the production of PersL. The presented here results of a detailed analysis of traps based on the T_max_-T_stop_ method in correlation with structural changes—particularly site occupancy—will, in our opinion, help to establish the relationship between structural changes in crystals and their effect on traps creation and redistribution.

## 2. Materials and Methods

### 2.1. Materials Preparation

Y_3_Al_1.99_Cr_0.01_Ga_3_O_12_ (YAGG:Cr^3+^) nanophosphors were synthesized by using a modified Pechini method described previously in more detail [19]. Y_2_O_3_ (99.999% purity, Stanford Materials Corporation, Lake Forest, IL, USA ), AlCl_3_ (99.999% purity, Alfa Aesar, Haverhill, MA, USA) GaCl_3_ (99.999% purity, Sigma-Aldrich, Saint Louise, MO, USA), and Cr(NO_3_)_3_×9H_2_O (99.99% purity, Alfa Aesar, Haverhill, MA, USA), additionally Citric acid (99.5% purity, Alfa Aesar, Haverhill, MA, USA) aqueous solution and ethylene glycol (99% purity, POCH. S.A., Basic, Gliwice, Poland) were used as starting materials. The gel was subsequently annealed at selected temperatures from 800 to 1300 °C for 16 h in static air for further investigations.

### 2.2. Characterization Techniques

*X-ray powder diffraction* (XRPD) patterns were acquired by a PANalytical X’Pert pro X-ray (Malvern Pananalytical, Malvern, UK) powder diffractometer at 40 kV and 30 mA in the 2θ range of 10–80° (2θ step: 0.02626°) using nickel-filtered Cu K_α1_ radiation. The phase identification was performed using the X’pert HighScore Software. The phase composition, cell parameters, crystallite sizes, and microstrain as the occupancy of the octahedral and tetrahedral sites in the YAGG structures were evaluated based on the Rietveld method [20] using the WinPLOTR, and WinPLOTR-2006 applications. The average crystallite size was calculated using a Williamson–Hall analysis.

*Photoluminescence emission* (PL) and *excitation* (PLE) spectra were measured using the FLS980 Fluorescence Spectrometer (Edinburgh Instruments Ltd., Livingston, UK) equipped with a 450 W Xenon lamp as an excitation source. The excitation arm was supplied with a holographic grating of 1800 lines/mm, blazed at 300 nm, while the emission arm was supplied with ruled grating, 1800 lines/mm blazed at 750 nm. Both the excitation and emission monochromators were in the Czerny-Turner configuration. The photomultiplier tube R928P (Hamamatsu Photonics, Shizuoka, Japan) was used as a detector. The scanning range was from 250 to 680 nm for the PLE spectrum and from 460 to 820 nm for the PL spectrum with a spectral resolution of 0.2 nm. All spectra were corrected for the sensitivity of detectors and intensity excitation source.

*The thermoluminescence* (TL) glow curves were collected with a lexygresearch–Fully Automated TL/OSL Reader (Freiberg Instruments GmbH, Freiberg, Germany) from room temperature to 300 °C with heating rates 2, 1, 0.75, 0.5, and 0.25 °C·s^−1^. Varian VF-50J/S RTG tube (Varian Medical Systems Inc., Palo Alto, CA, USA) with tungsten core and copper case as an X-ray radiation source was used as an irradiation source. The voltage and amperage for the X-Ray source were 15 kV and 0.1 mA respectively. The signal was collected using PMT R13456 (Hamamatsu Photonics, Shizuoka, Japan Shizuoka, Japan) with a filter 721/65 Brighline HC (Semrock Inc., Rochester, NY, USA). Powder samples were prepared for measurements the same as was described in our previous work [17] For detailed analyses of the traps redistribution as an annealing temperature function, the T_max_-T_stop_ (partial cleaning) experiment proposed by McKeever was performed [21]. The sample was irradiated at room temperature with the following partially heating to a temperature T_stop_ and cooling back to room temperature (25 °C). TL curves were recorded from room temperature to 300 °C with a heating rate of 0.5 °C·s^−1^ and integration (detection) time of 0.1 s. Positions first maximum T_max_ in the glow-curve versus T_stop_ are plotted in the T_stop_ range between 30 and 200 °C with step 10 °C to cover completely the glow curve.

## 3. Results and Discussion

### 3.1. Microstructure

**Figure 1:**
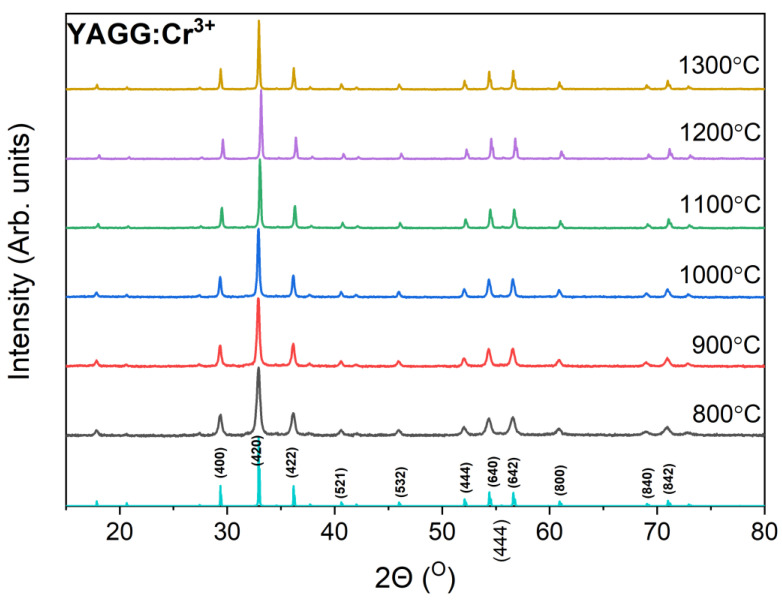
XRPD pattern of the YAGG:Cr^3+^ nanopowders annealed at different temperatures.

Figure 1 shows the XRPD patterns of the YAGG:Cr^3+^ powders annealed at different temperatures. The XRPD patterns were refined by Rietveld analysis (see Figure S1), which showed the presence of pure Y_3_Al_2_Ga_3_O_12_ phase of the cubic *I*$\bar{a}$3*d* space group [22]. The crystallographic data and refinement details of the structure including lattice parameter and grain size, are summarized in Table 1.

**Figure 2:**
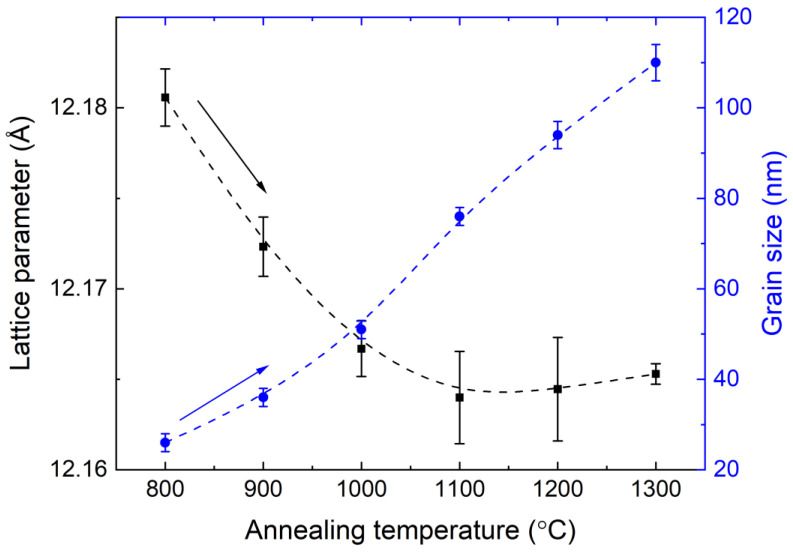
The influence of the annealing temperature on the grain size and lattice parameter of the YAGG nanopowders.

Figure 2 presents the calculated values of grain sizes and lattice parameters of the YAGG:Cr^3+^ nanopowders for the sample annealed at various temperatures. Direct dependence of the average grain size on temperature is clearly seen, increasing the temperature from 800 °C to 1300 °C led to a growth in the average crystallite size from 26(2) nm to 110(2) nm. Meanwhile, the lattice parameters decrease from 12.1806(16) Å to 12.1640(26) Å for an annealing temperature of 1100 °C, and a further rise in the temperature barely changes the lattice parameter.

The change in the lattice parameter can be explained by the influence of the surface. The YAGG:Cr^3+^ nanopowders with an average crystalline size above 50 nm do not reveal any structural alteration, while the samples smaller than 50 nm show an increase in the lattice parameter. The noticed decrease in its value is most probably related to the increase in crystallite size through the repulsive force at the surface due to unpaired electronic orbitals. These dipoles repel each other, which reduces the value of the equilibrium lattice parameter for the smaller crystallites (with a higher surface-to-volume ratio) to be greater than that of crystallites with larger sizes [23]. This is also reflected in the microstrain, which decreases with increasing crystallite size (Figure S2).

**Figure 3:**
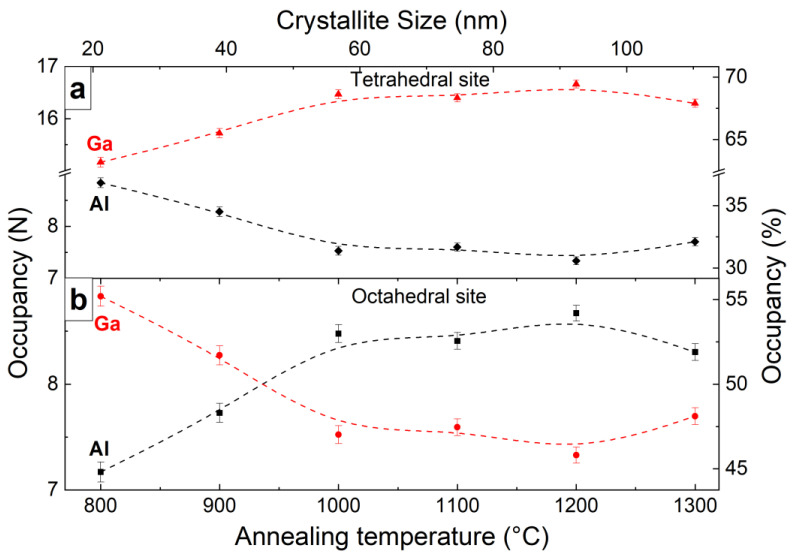
The influence of the annealing temperature on the occupancy of the tetrahedral (**a**) octahedral (**b**) sites of the Y_3_Al_2_Ga_3_O_12_ nanopowders.

The general formula of the YAGG crystal structure can be represented as C_3_A_2_D_3_O_12_, where the C–dodecahedral site is occupied by Y^3+^ ions, while A, D-octahedral, and tetrahedral sites, respectively, are occupied by Al^3+^ and Ga^3+^ ions [24]. Unlike Al^3+^ and Ga^3+^ ions, Cr^3+^ can occupy only octahedral sites, because the crystal field stabilization energy of tetravalent chromium ions in tetrahedral coordination is about a third of the value in octahedral coordination [25]. Otherwise, Cr^4+^ can occupy both octahedral and tetrahedral sites [26]. Accordingly, the only variable independent parameter is the occupancy of octahedral and tetrahedral sites by Al^3+^ and Ga^3+^ ions. The increase in annealing temperature leads to redistributions of Ga^3+^ and Al^3+^ ions between the different sites in the garnet lattice. Figure 3 shows the change in the occupancy of the octahedral and tetrahedral sites of the samples at different annealing temperatures. It is interesting to recall the fact that only a third of Ga^3+^ is in the octahedral site, and the rest–is in the tetrahedral [23].

An important parameter of YAGG materials is the fractional parameter ***f***_Ga_, which indicates the degree preference of the tetrahedral site by Ga^3+^. The fractional parameter ***f***_Ga_ was calculated according to the methodology described earlier [27]. It should be noted that the ***f***_Ga_ value higher than 0.6 indicates prefer occupation of the tetrahedral site by Ga^3+^ ions (see Figure S2). The calculated ***f***_Ga_ value was in the range of 0.63(1) to 0.70(1) and depended on crystalline size (Table 1). This indicates that the Ga^3+^ preferentially occupies the tetrahedral position. The tendency to occupy the tetrahedral site cannot be explained by cation sizes, as larger cations (Ga^3+^, 0.61 Å, and 0.47 Å in A and D sites, respectively [28]) tend to occupy the smaller tetrahedral positions, while the smaller cations (Al^3+^, 0.53 Å, and 0.39 Å in A and D sites respectively [29]) tend to occupy the larger octahedral site [27].

The decrease in the average grain size of the crystallites leads to a change in Ga^3+^ ions distributions resulting in a decrease ***f***_Ga_ from 0.69(1) at crystallite size 51(2) nm to ***f****_Ga_* 0.63(1) at crystallite size 26(2) nm (Figure S2) which is below the value ***f***_Ga_ (0.68–0.72) reported earlier for a single crystal of the same composition [27,29]. The decrease in the ***f***_Ga_ is probably caused by an increase in the lattice parameter of YAGG nanopowders caused by a decrease in the crystallite size. Beforehand the same results were detected on Y_3_Al_5-x_Ga_x_O_12_ single crystals where the increase in lattice parameter caused a decrease of fractional parameter ***f***_Ga_. The Ga^3+^ cation distributions in Y_3_Al_2_Ga_3_O_12_ nanopowders are due to the binding nature of the cation-oxygen bonds and cation-cation repulsive forces. Most likely, the peculiar distribution of cations in the Y-Al-Ga system is associated with the compensation of the cation-cation repulsive force, as well as the effect of electronegativity [27].

### 3.2. Photoluminescence

**Figure 4:**
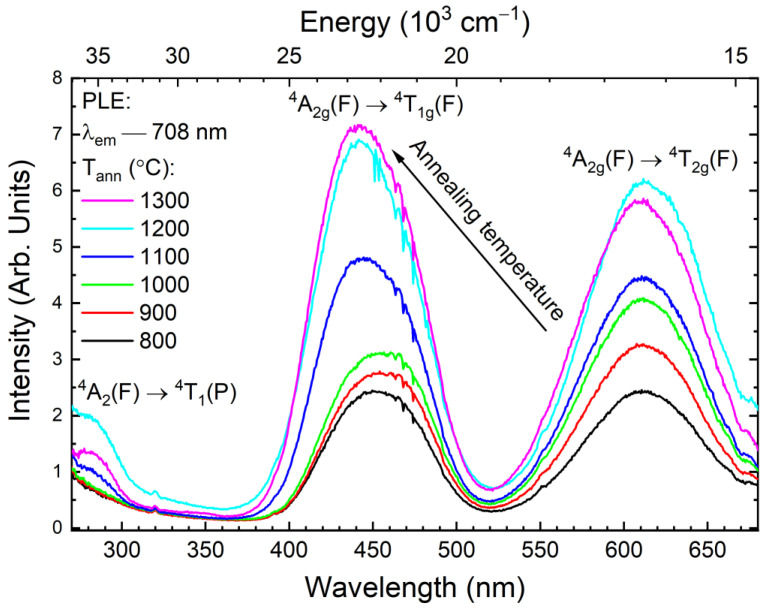
Photoluminescence excitation spectra of the YAGG doped with 1% Cr^3+^ powders as a function of annealing temperature.

**Figure 5:**
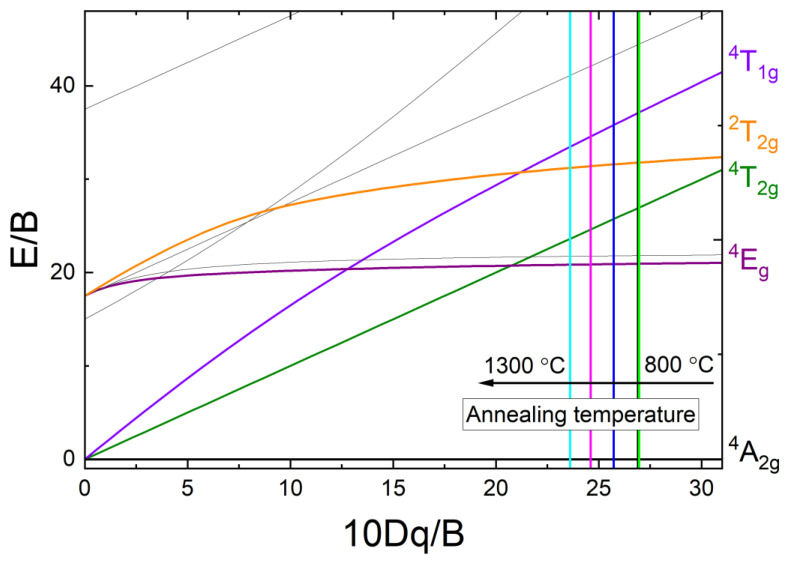
d^3^ Tanabe–Sugano diagram for Cr^3+^ ion in an octahedral environment. Vertical solid lines indicate the calculated values of 10Dq/B parameter for samples annealed at different temperatures. The line colors correspond to the colors of the plotted PLE spectra.

A comparison of the PLE spectra of Cr^3+^ ions in YAGG samples annealed at different temperatures from 800 °C to 1300 °C reveals the presence of a blue shift of the ^4^A_2g_(F) → ^4^T_1g_(F) transition. It can be noted that the maximum of that band shifts its position on the energy scale from 22 779 cm^−1^ to 23 031 cm^−1^ (ΔE ~ 250 cm^−1^). In contrast, the band assigned as ^4^A_2g_(F) → ^4^T_2g_(F) almost does not change its position (16 385 cm^−1^) even for the highest annealing temperature. This effect is visible in Figure 4. It should be noted that the abnormal behavior of the band ^4^T_1g_ is observed, namely the appearance of inflexion on the shoulder from the lower energies side. This anomaly may be associated with the presence of the band ^2^T_2g_ (Figure S3). The position of this inflexion coincides with the corresponding line in the Sugano-Tanabe diagram (Figure 5) [30].

As the Cr^3+^ ion belongs to the transition metal ions, its spectroscopic properties depend much more on the crystal field strength than is the case for, e.g., lanthanide ions. For example, in the presence of a low field, the Cr^3+^ luminescence occurs from the spin-allowed ^4^T_2g_ → ^4^A_2_ transition (broadband emission with a short lifetime), but for a high crystal field (more than 2.1), the dominant luminescence originates from the spin-forbidden ^2^E→ ^4^A_2_ transition, which results in the presence of sharp zero phonon R lines, a phonon sideband, and a longer decay time [30,31]. In the YAGG host, the Cr^3+^ ion substitutes the Al^3+^ site with the octahedral symmetry, so it is possible to determine the values of crystal field parameters by using a Tanabe-Sugano diagram for a d^3^ system [30]. The Racah parameter B, as well as the ligand field splitting term Dq for each annealing temperature, were found by determining the positions of the two excitation peaks evident in Figure 4, calculating their energy ratio, and locating these ratios on the proper Tanabe-Sugano diagram [32]. The result of this procedure is presented in Figure 5. The calculated ratios increase with the temperature from 1.378 to 1.417 and obtained 10Dq/B parameters decrease from 27.203 to 23.730, respectively. Hence, we can determine the values of the parameter B as the point of intersection of the vertical line representing 20 Dq/B, with the term line of the lowest ^4^T_2g_(F) state on the Tanabe-Sugano diagram. Finally, using the calculated Racah parameter B, one can determine the Dq value representing the crystal field strength for this system as well as the β = B/B_0_ (where B_0_ is the Racah parameter for free Cr^3+^ ion, equal to 1030 cm^−1^). All the calculated parameters are presented in Table 2.

The slightly decreasing distance between ^4^T_1g_(F) and ^4^T_2g_(F) terms over the range of temperatures investigated indicates a decreasing repulsion interaction of the d electrons and, hence, an expansion of the d electrons cloud. This effect is called the nephelauxetic effect [33]. In addition, with the increase of annealing temperature (and therefore with the crystallite size) an increase of the β parameter, determining the degree of bonding covalence is observed. In the case of the investigated YAGG:1%Cr^3+^ sample, the bonding covalence degree appears to decrease with the crystallite size. As can be seen from the data obtained, the YAGG:Cr^3+^ samples annealed at lower temperatures (between 800 to 1000 °C) seem to be identical in terms of crystal field calculations. Thereafter, as the annealing temperature increases, the Dq/B parameter starts to decrease and reaches a minimum (~24) at 1200 °C. However, despite the observed tendency for higher temperatures to decrease the crystal field intensity with crystallite size, a breakdown is observed at 1300 °C. At this temperature, the redistribution of ions does not change compared to the sample annealed at 1100 °C. This tendency is similar to the data for the lattice parameter and Ga^3+^ and Al^3+^ sites occupancy.

**Figure 6:**
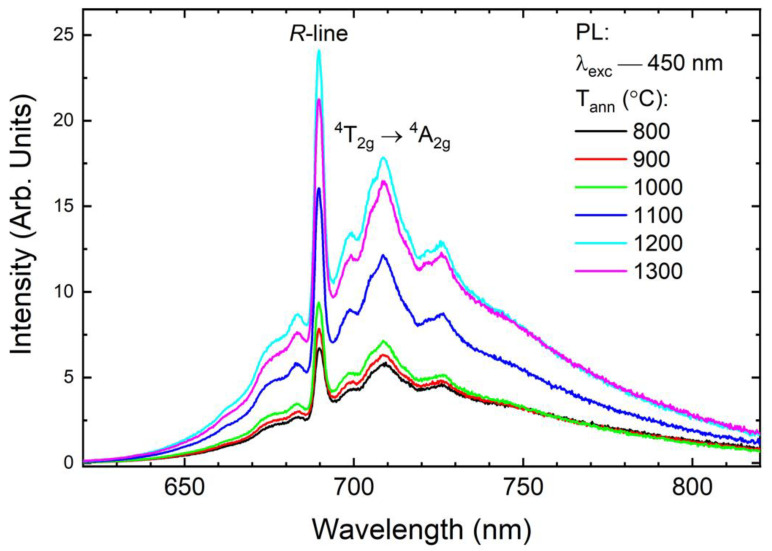
Photoluminescence spectra of the YAGG: 1% Cr nanopowders as a function of annealing temperature. Excitation 450 nm, at room temperature.

The PL spectra of samples annealed at temperatures from 800 to 1300 °C are shown in Figure 6. The sharp and strong PL peak at ∼690 nm, is related to the ^4^A_2g_ → ^2^E_g_ transition, while the broadband is related to the ^4^T_2g_ → ^4^A_2g_ transition.

It was reported elsewhere, that the PL intensity of the Cr-doped garnet may strongly depend on the particle size [34]. In this work, particularly for samples annealed at temperatures from 800 to 1000 °C, the PL intensity has almost the same intensity. This is consistent with the reported structural features-for these samples, the average particle size is found to be almost the same. However, for lower annealing temperatures a significant number of O–H groups remain on the particle surfaces or in the pores and it can lead to luminescence quenching [35]. With a further increase in annealing temperature, an increase in intensity is also observed, which reaches its maximum value for a temperature of 1200 °C and then remains unchanged.

### 3.3. Thermoluminescence Analysis

**Figure 7:**
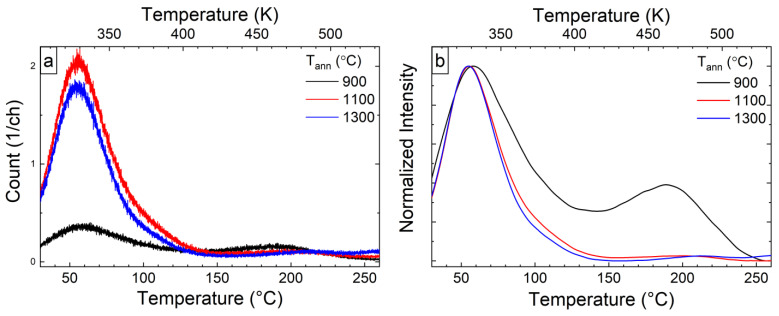
TL glow curves as it is (**a**) and normalized (**b**), detected after irradiation with X-rays for 5 min, β–0.5 °C/s.

To determine the effect of structural changes on the traps formation and redistribution, the TL curves for all investigated powders after irradiation by X-ray were recorded and analyzed. The first and dominant TL peak for the YAGG:Cr^3+^ sample is observed at around 60 °C (Figure 7). Furthermore, a second high-temperature peak with a maximum at 190 °C is more pronounced at lower annealing temperatures. In YAGG:Cr^3+^ annealed at 900 °C, the two TL peaks reflect two well-defined series of traps. As the annealing temperature increases, the intensity of the second peak decreases slightly and it becomes less prominent compared to the first peak, whose intensity increases. Importantly, both peaks have a similar symmetrical shape, suggesting that they may be the result of a superposition of a series of traps with a continuous energies distribution [21,36].

**Figure 8:**
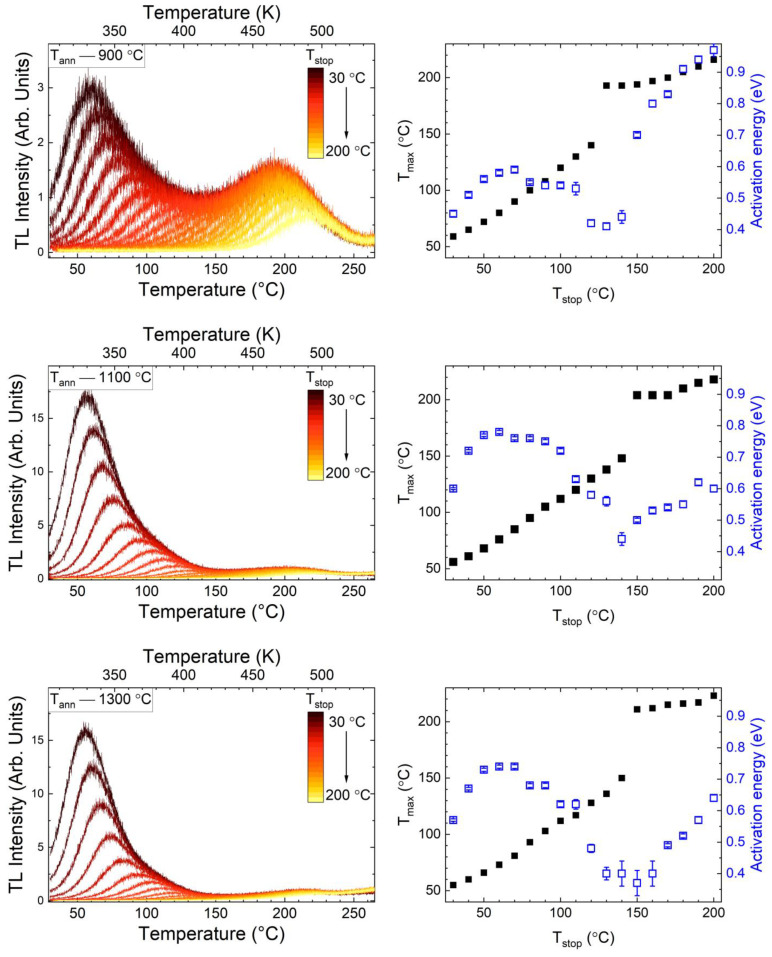
TL glow curves were detected with the T_max_-T_stop_ methods and their analysis using the Initial-Rise method (blue points in the right row of panels). Samples were charged with X-rays for 5 min, β—0.5 °C/s.

As can be seen from the results of structural studies carried out for the samples doped with chromium, three critical temperatures can be distinguished for the powders studied, namely 900, 1100, and 1300 °C. At 900 °C, crystal structure formation was observed for the garnet synthesis by the Pechini method [17]. The temperature of 1100 °C was chosen based on the minimum values of the lattice parameter and the particle size, which is still less than 100 nm. The third is 1300 °C, at which the lattice parameter begins to increase and the crystal field decreases. To further analyze the traps contributing to the main thermoluminescence peaks, the T_max_-T_stop_ method was carried out for selected annealing temperatures (Figure 8 left panel). In addition, the Initial Rise method [37] was used to determine the energy from each of the recorded curves. Using data obtained, plots of the dependence of T_max_ (black square) and activation energy (blue square) on T_stop_ value were drawn for observed traps, which are presented in Figure 8 (right panel).

Two maxima are clearly evident in the plots, which are of different natures. The first maximum is linear with a slope near unity, indicating a series of first-order peaks with a quasi-continuous distribution of peaks (and, therefore, of trapping centers) [38,39]. The second maximum consists of a single first-order peak only in the T_max_-T_stop_ part of the curve (150–200 °C) and in the further course is simply a line with zero slope [38]. This is because the peak has not shifted as the initial population of trapped charges decreases. From the point of view of the activation energy, the first maximum in all samples is the same and is about 0.75 ± 0.02 eV, which is close to the value observed previously for the same matrix with different co-dopants [17,40]. In contrast, the activation energy of the second maximum noticeably decreased from about 0.9 to 0.5 eV with increasing an annealing temperature. The invariability of the position of the maximum with decreasing activation energy can be explained by the following Equation (1):

(1) $$\frac{\beta E}{kT_{m}^{2}}=s\cdot \exp\left\{-\frac{E}{kT_{m}}\right\}$$

where *T*_m_ is the glow curve maximum, *E* is the activation energy, *β* is the heating rate (in K/s), and *s*—frequency factor (s^−1^). Equation (1) shows that the frequency factor also plays an important role in the de-trapping process. As the activation energy of the second maximum decreases from 0.9 to 0.5 eV, the value of the frequency factor decreases from 10^8^ to 10^3^ s^−1^, and the process of releasing trapped electrons occurs more slowly [37,41]. Finally, it is likely that one TL peak with a position at a higher *T*_m_ may have a lower activation energy than the previous one. Combining these results with the previous analysis of the structure and optical properties, it can be concluded with a high probability that the traps with lower energy values are related to changes occurring inside the phosphors. In particular, morphology, phase change, redox reactions of the dopant ions, changes due to clustering of such dopants, etc. Specifically, one of the limiting points may be –OH groups, which may be present in samples after lower annealing temperatures [35] and which can transform into oxygen vacancies as a result of annealing at higher temperatures.

## 4. Conclusions

It was found, that increasing the annealing temperature to 1100 °C leads to a decrease in the lattice parameter to 12.164, after which it remains almost unchanged. In parallel, a redistribution of the Ga^3+^ and Al^3+^ ions between octa- and tetrahedral sites in the garnet lattice occurs. Above 1100 °C, most Ga^3+^ ions (up to 70%) remain in the tetrahedral site while Al ions (up to 55%) move to the octahedral site. These structural changes correlate with the optical characteristics of both PL and PersL. As the grain size increases, the electron-phonon coupling decreases, whereas the crystal-field strength increases. A similar trend is observed for thermoluminescence curves. The maximum of the TL intensity reached 1100 °C and remains unchanged for higher temperatures.

It can therefore be concluded that the higher annealing temperature (>1100 °C) did not improve the optical properties of studied phosphors. Consequently, the YAGG phosphor annealed at 1100 °C, showing an acceptable degree of particle agglomeration, may already be suitable for practical applications as starting materials for the production of, among others, high-quality optical ceramics and luminescent markers for imaging in the first biological window.

## Figures and Tables

**Table 1:** Cell parameter ***a***, crystallite size ***D***_XRPD_, lattice microstrain ***ε***, an average number of Ga^3+^ ions per lattice in octahedral site ***N***(A) and tetrahedral site ***N***(D), and fractional parameter ***f***_Ga_ obtained by applying the Rietveld method to the XRPD patterns.

Annealing Temperature, °C	*a*, Å	*D*_XRPD_, nm	*ε*	*N*(A)	*N*(D)	*f*_Ga_
800	12.1806(16)	26(2)	0.00223(10)	8.83(9)	15.16(9)	0.632(7)
900	12.1723(16)	36(2)	0.00159(9)	8.27(9)	15.72(9)	0.655(7)
1000	12.1667(15)	51(2)	0.00113(8)	7.52(9)	16.47(9)	0.687(8)
1100	12.1640(26)	76(2)	0.00077(6)	7.59(8)	16.40(8)	0.684(7)
1200	12.1645(29)	94(2)	0.00061(7)	7.33(8)	16.66(8)	0.695(7)
1300	12.1653(6)	110(2)	0.00053(4)	7.70(8)	16.30(8)	0.679(7)

**Table 2:** Crystal field parameters for YAGG:Cr^3+^ nanopowders as a function of annealing temperature calculated with Tanabe-Sugano diagram for a d^3^ system.

Annealing Temperature, °C	υ_2_/υ_1_ Ratio	Dq/B	B_2_, cm^−1^	β = B2/B0	Crystallite Size *D*_XRPD_, nm
800	1.378	27.203	828.889	0.805	26(2)
900	1.378	27.203	828.889	0.805	36(2)
1000	1.378	27.203	828.889	0.805	51(2)
1100	1.391	26.045	874.601	0.849	76(2)
1200	1.417	23.730	980.021	0.951	94(2)
1300	1.404	24.887	925.828	0.899	110(2)

## Data Availability

The data presented in this study are available on request from the corresponding author.

## Supplementary Materials

The following supporting information can be downloaded at: https://www.mdpi.com/article/10.3390/ma15134407/s1, Figure S1: X-ray diffraction patterns with main (*hkl*) of the samples. The results of the Rietveld refinement analysis; Figure S2: The influence of the annealing temperature on the microstrain and fractional ***f***_Ga_ parameters of the Y_3_Al_2_Gd_3_O_12_ powders calculated from X-ray diffraction patterns; Figure S3: Deconvolution of the PLE spectra of the YAGG:Cr^3+^ nanophosphors. Reference [20] is cited in the Supplementary Materials.

## References

[1] Xu J., Tanabe S. (2019). Persistent Luminescence Instead of Phosphorescence: History, Mechanism, and Perspective. J. Lumin..

[2] Poelman D., Van der Heggen D., Du J., Cosaert E., Smet P.F. (2020). Persistent Phosphors for the Future: Fit for the Right Application. J. Appl. Phys..

[3] Van den Eeckhout K., Smet P.F., Poelman D., Van den Eeckhout K., Smet P.F., Poelman D. (2010). Persistent Luminescence in Eu^2+^-Doped Compounds: A Review. Materials.

[4] Bessière A., Sharma S.K., Basavaraju N., Priolkar K.R., Binet L., Viana B., Bos A.J.J., Maldiney T., Richard C., Scherman D. (2014). Storage of Visible Light for Long-Lasting Phosphorescence in Chromium-Doped Zinc Gallate. Chem. Mater..

[5] Hölsä J. (2009). Persistent Luminescence Beats the Afterglow: 400 Years of Persistent Luminescence. Electrochem. Soc. Interface.

[6] Hossain M.K., Hossain S., Ahmed M.H., Khan M.I., Haque N., Raihan G.A. (2021). A Review on Optical Applications, Prospects, and Challenges of Rare-Earth Oxides. ACS Appl. Electron. Mater..

[7] Hossain M.K., Ahmed M.H., Khan M.I., Miah M.S., Hossain S. (2021). Recent Progress of Rare Earth Oxides for Sensor, Detector, and Electronic Device Applications: A Review. ACS Appl. Electron. Mater..

[8] Castaing V., Arroyo E., Becerro A.I., Ocaña M., Lozano G., Míguez H. (2021). Persistent Luminescent Nanoparticles: Challenges and Opportunities for a Shimmering Future. J. Appl. Phys..

[9] Xu J., Ueda J., Tanabe S. (2017). Toward Tunable and Bright Deep-Red Persistent Luminescence of Cr^3+^ in Garnets. J. Am. Ceram. Soc..

[10] Algar W.R., Massey M., Rees K., Higgins R., Krause K.D., Darwish G.H., Peveler W.J., Xiao Z., Tsai H.Y., Gupta R. (2021). Photoluminescent Nanoparticles for Chemical and Biological Analysis and Imaging. Chem. Rev..

[11] Xu J., Ueda J., Zhuang Y., Viana B., Tanabe S. (2015). Y_3_Al_5−x_ Ga_x_O_12_:Cr^3+^: A Novel Red Persistent Phosphor with High Brightness. Appl. Phys. Express.

[12] Katayama Y., Viana B., Gourier D., Xu J., Tanabe S. (2016). Photostimulation Induced Persistent Luminescence in Y_3_Al_2_Ga_3_O_12_:Cr^3+^. Opt. Mater. Express.

[13] Ueda J., Xu J., Takemura S., Nakanishi T., Miyano S., Segawa H., Tanabe S. (2021). How Many Electron Traps Are Formed in Persistent Phosphors?. ECS J. Solid State Sci. Technol..

[14] Fedyk R., Hreniak D., Łojkowski W., Strek W., Matysiak H., Grzanka E., Gierlotka S., Mazur P. (2007). Method of Preparation and Structural Properties of Transparent YAG Nanoceramics. Opt. Mater..

[15] Dai Z., Boiko V., Grzeszkiewicz K., Saladino M.L., Li J., Hreniak D. (2020). Effect of Annealing Treatment on the Persistent Luminescence of Y_3_Al_2_Ga_3_O_12_:Ce^3+^,Cr^3+^,Pr^3+^ Ceramics. Opt. Mater..

[16] Xu J., Murata D., Ueda J., Viana B., Tanabe S. (2018). Toward Rechargeable Persistent Luminescence for the First and Third Biological Windows via Persistent Energy Transfer and Electron Trap Redistribution. Inorg. Chem..

[17] Boiko V., Dai Z., Markowska M., Leonelli C., Mortalò C., Armetta F., Ursi F., Nasillo G., Saladino M.L., Hreniak D. (2021). Particle Size-Related Limitations of Persistent Phosphors Based on the Doped Y_3_Al_2_Ga_3_O_12_ System. Sci. Rep..

[18] Pei P., Chen Y., Sun C., Fan Y., Yang Y., Liu X., Lu L., Zhao M., Zhang H., Zhao D. (2021). X-ray-Activated Persistent Luminescence Nanomaterials for NIR-II Imaging. Nat. Nanotechnol..

[19] Boiko V., Zeler J., Markowska M., Dai Z., Gerus A., Bolek P., Zych E., Hreniak D. (2019). Persistent Luminescence from Y_3_Al_2_Ga_3_O_12_ Doped with Ce^3+^ and Cr^3+^ after X-ray and Blue Light Irradiation. J. Rare Earths.

[20] Young R.A. (1995). The Rietveld Method.

[21] McKeever S.W.S. (1985). Thermoluminescence of Solids.

[22] Chaika M., Paszkowicz W., Strek W., Hreniak D., Tomala R., Safronova N., Doroshenko A., Parkhomenko S., Dluzewski P., Kozłowski M. (2019). Influence of Cr Doping on the Phase Composition of Cr,Ca:YAG Ceramics by Solid State Reaction Sintering. J. Am. Ceram. Soc..

[23] Ayyub P., Palkar V.R., Chattopadhyay S., Multani M. (1995). Effect of Crystal Size Reduction on Lattice Symmetry and Cooperative Properties. Phys. Rev. B.

[24] Chaika M.A., Dulina N.A., Doroshenko A.G., Parkhomenko S.V., Gayduk O.V., Tomala R., Strek W., Hreniak D., Mancardi G., Vovk O.M. (2018). Influence of Calcium Concentration on Formation of Tetravalent Chromium Doped Y_3_Al_5_O_12_ Ceramics. Ceram. Int..

[25] Dereń P.J., Malinowski M., Strȩk W. (1996). Site Selection Spectroscopy of Cr^3+^ in MgAl_2_O_4_ Green Spinel. J. Lumin..

[26] Chaika M.A., Tomala R., Strek W., Hreniak D., Dluzewski P., Morawiec K., Mateychenko P.V., Fedorov A.G., Doroshenko A.G., Parkhomenko S.V. (2019). Kinetics of Cr^3+^ to Cr^4+^ Ion Valence Transformations and Intra-Lattice Cation Exchange of Cr^4+^ in Cr,Ca:YAG Ceramics Used as Laser Gain and Passive Q-Switching Media. J. Chem. Phys..

[27] Nakatsuka A., Yoshiasa A., Yamanaka T. (1999). Cation Distribution and Crystal Chemistry of Y_3_Al_5−X_Ga_x_O_12_ (0 ≤ x ≤ 5) Garnet Solid Solutions. Acta Crystallogr. Sect. B Struct. Sci..

[28] Marezio M., Remeika J.P., Dernier P.D. (1968). IUCr Cation Distribution in Y_3_Al_5−c_Ga_c_O_12_ Garnet. Acta Crystallogr. Sect. B Struct. Sci. Cryst. Eng. Mater..

[29] Shannon R.D. (1976). Revised Effective Ionic Radii and Systematic Studies of Interatomic Distances in Halides and Chalcogenides. Acta Crystallogr. Sect. A.

[30] Adachi S. (2021). Review—Photoluminescence Properties of Cr^3+^-Activated Oxide Phosphors. ECS J. Solid State Sci. Technol..

[31] Shen Y., Bray K. (1997). Effect of Pressure and Temperature on the Lifetime of in Yttrium Aluminum Garnet. Phys. Rev. B—Condens. Matter Mater. Phys..

[32] Tanabe Y., Sugano S. (1954). On the Absorption Spectra of Complex Ions II. J. Phys. Soc. Japan.

[33] Jørgensen C.K., Cotton F.A. (1962). The Nephelauxetic Series. Progress in Inorganic Chemistry.

[34] Głuchowski P., Małecka M., Stręk W., Ryba-Romanowski W., Solarz P. (2017). Size Effect in Novel Red Efficient Garnet Nanophosphor. J. Phys. Chem. C.

[35] Dai Z., Boiko V., Grzeszkiewicz K., Markowska M., Ursi F., Hölsä J., Saladino M.L., Hreniak D. (2020). Effect of Annealing Temperature on Persistent Luminescence of Y_3_Al_2_Ga_3_O_12_:Cr^3+^ Co-Doped with Ce^3+^ and Pr^3+^. Opt. Mater..

[36] Van den Eeckhout K., Bos A.J.J., Poelman D., Smet P.F. (2013). Revealing Trap Depth Distributions in Persistent Phosphors. Phys. Rev. B.

[37] Bos A.J.J. (2007). Theory of Thermoluminescence. Radiat. Meas..

[38] McKeever S.W.S. (1980). On the Analysis of Complex Thermoluminescence. Glow-Curves: Resolution into Individual Peaks. Phys. Status Solidi.

[39] Sójka M., Zeler J., Zych E. (2021). Effect of Ge:Si Ratio and Charging Energy on Carriers Trapping in Y_2_(Ge,Si)O_5_:Pr Powders Observed with Thermoluminescence Methods. J. Alloys Compd..

[40] Yang T., Jiang H., Hai O., Dong Y., Liu S., Gao S. (2021). Effect of Oxygen Vacancies on the Persistent Luminescence of Y_3_Al_2_Ga_3_O_12_:Ce^3+^,Yb^3+^Phosphors. Inorg. Chem..

[41] Magdalene Mashangva M., Nara Singh T.B.S. (2011). Estimation of Optimal Trapping Parameters Relevant to Persistent Luminescence. Indian J. Pure Appl. Phys..

