# Supplementary material for: Size-Dependent Persistent Luminescence of YAGG:Cr3+ Nanophosphors
Source: Materials (Basel). 2022 Jun 22;15(13):4407. doi: 10.3390/ma15134407 (PMC9267483; doi:10.3390/ma15134407)
Supplement: Supplementary file 1 [file materials-15-04407-s001.zip › materials-1774096-supplementary.pdf]

Supplementary Materials

Size-dependent persistent luminescence of YAGG:Cr^{3+} nanophosphors

Vitalii Boiko[1*], Zhengfa Dai[1,2], Mykhailo Chaika[1], Karina Grzeszkiewicz[1], Jiang Li[2,3], Wieslaw Strek[1] and Dariusz Hreniak[1]

1. Division of Optical Spectroscopy, Institute of Low Temperature and Structure Research, Polish Academy of Sciences, Okolna 2, PL-50-422, Wrocław, Poland

2. Key Laboratory of Transparent Opto-functional Inorganic Materials, Shanghai Institute of Ceramics, Chinese Academy of Sciences, Shanghai 201899, China.

3. Center of Materials Science and Optoelectronics Engineering, University of Chinese Academy of Sciences, Beijing 100049, China.

Correspondence: v.boiko@intibs.pl

The structure refinements were carried out by resorting to the FullProfSuite program and using the WinPLOTR, and WinPLOTR-2006 applications. The initial values for the parameters used in the present study were those of $Y_3Al_2Ga_3O_{12}$ nanopowders given by [20].

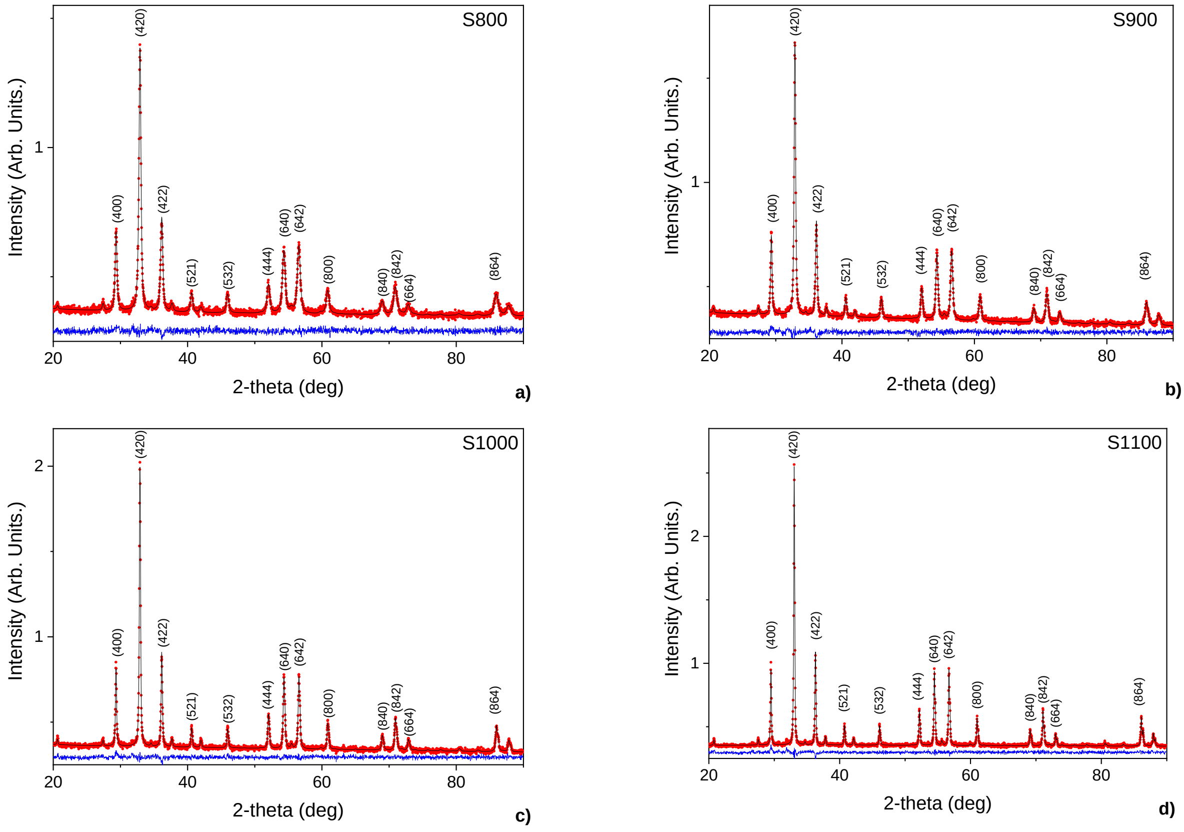

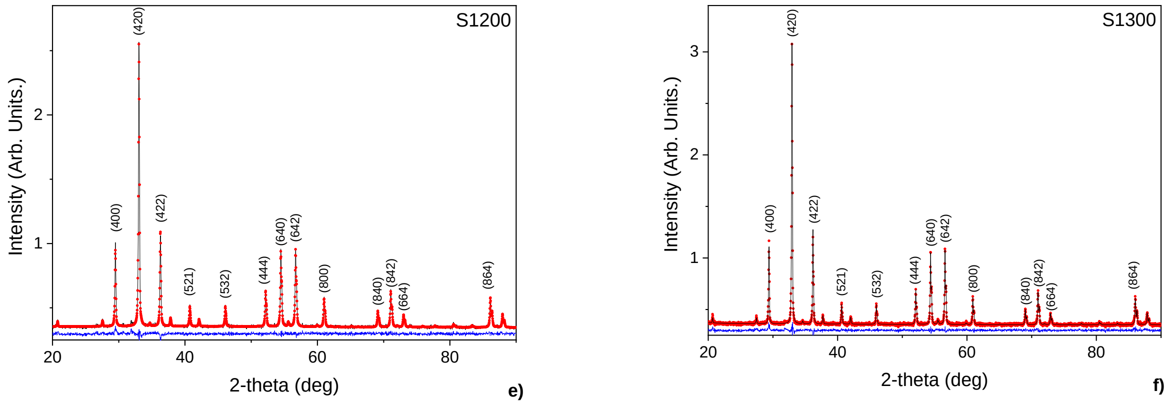

Figure S1. X-ray diffraction patterns with main (hkl) of the samples: a) S800, b) S900, c) S1000, d) S1100 e) S1200 f) S1300. The results of the Rietveld refinement analysis are shown by red and blue curve curves.

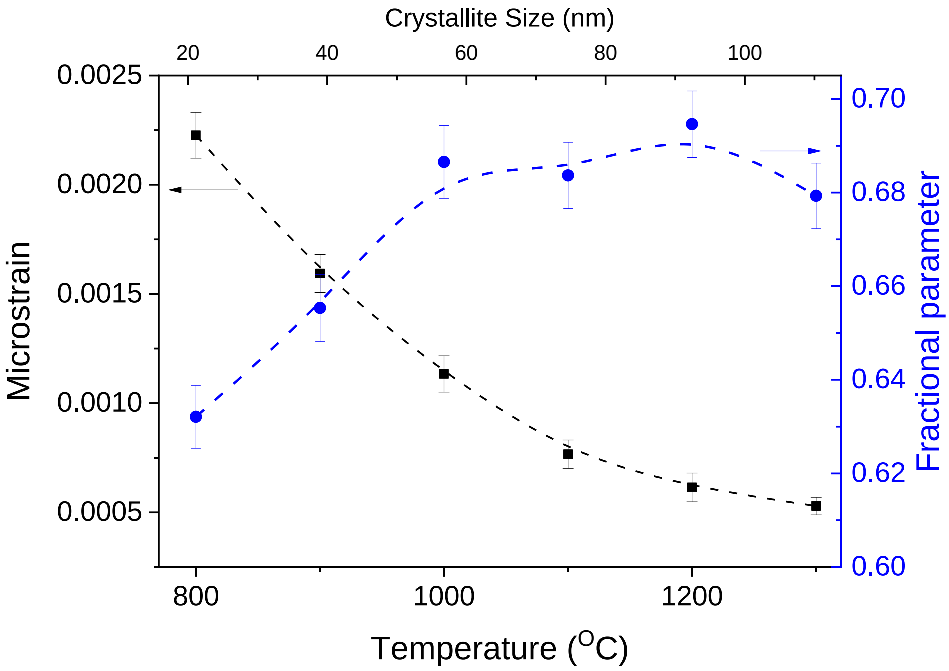

Figure S2. The influence of the annealing temperature on the microstrain and fractional f_{Ga} parameters of the $Y_3Al_2Ga_3O_{12}$ nanopowders calculated from X-ray diffraction patterns.

The structural formula in the $Y_3Al_2Ga_3O_{12}$ system can be generally expressed as $[Y]_3[Al_{1-p}Ga_p]_2[Al_{2p/3}Ga_{(3-2p)/3}]_3O_{12}$, where p is the occupancy parameter of Ga^{3+} ions on the octahedral site. The fractional parameter f_{Ga} corresponding to the degree of Ga^{3+} preference for the tetrahedral site is defined by $f_{Ga} = 1 - (2p/x)$. Ga^{3+} and Al^{3+} are distributed without any site preference on both the tetrahedral and the octahedral sites when f_{Ga} is 0.6 (p = 0.2x) because the cation ratio between the octahedral and the tetrahedral sites is 2:3. The f_{Ga} values for the compositions studied here are given in Table 1. The present f_{Ga} values are much larger than 0.6 for all the compositions, indicating that Ga^{3+} ions significantly prefer the tetrahedral site.

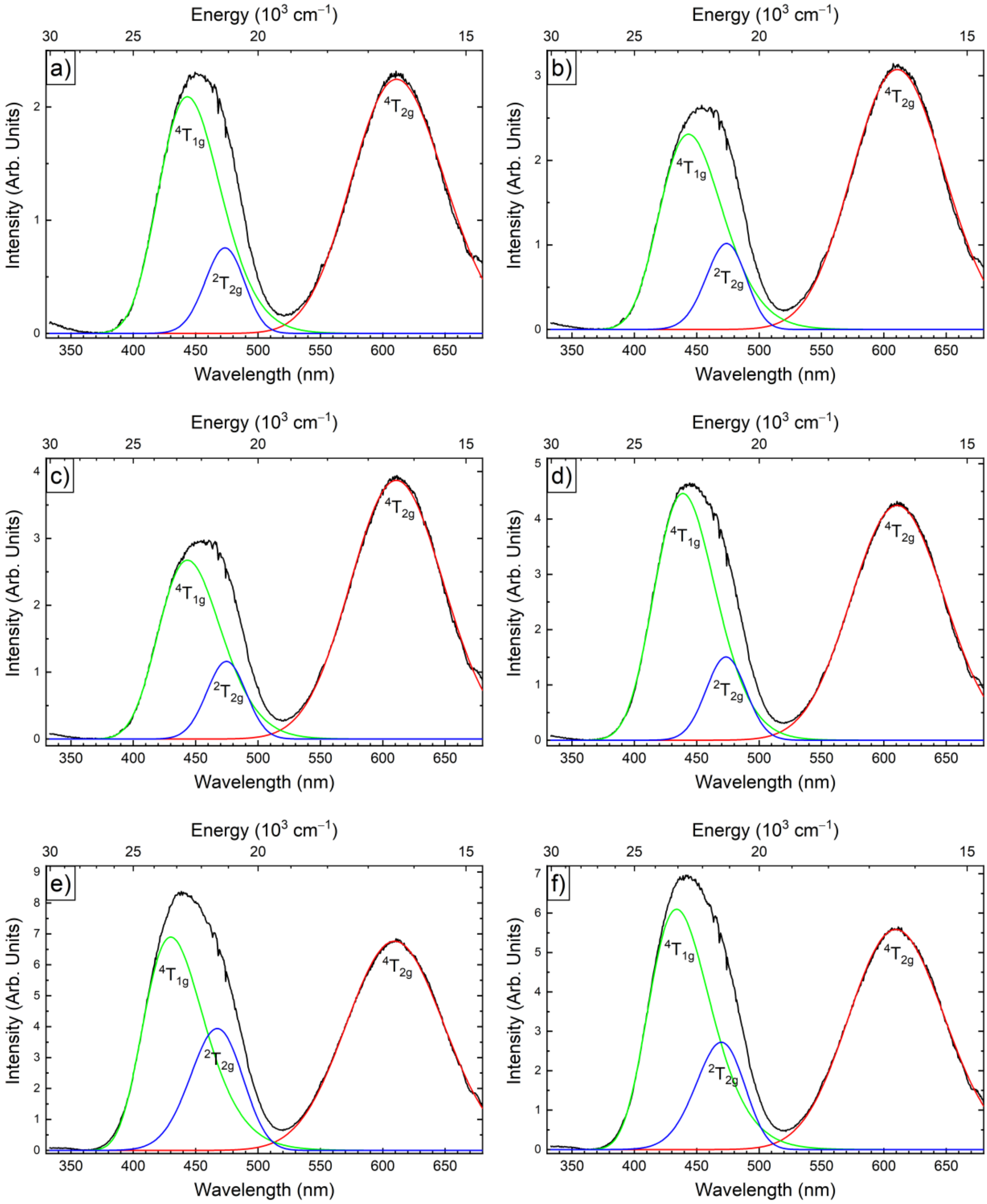

Figure S3. Deconvolution of the PLE spectra of the YAGG:Cr^{3+} nanophosphors annealed at a) 800 ˚C, b) 900 ˚C, c) 1000 ˚C, d) 1100 ˚C, e) 1200 ˚C, f) 1300 ˚C. The initial (raw) spectra are shown by a black line.
